# Supplementary material for: Discovery of 2-amide-3-methylester thiophenes that target SARS-CoV-2 Mac1 and repress coronavirus replication, validating Mac1 as an anti-viral target
Source: bioRxiv. 2023 Dec 27:2023.08.28.555062. Preprint. [Version 2] doi: 10.1101/2023.08.28.555062 (PMC10793406; doi:10.1101/2023.08.28.555062)
Supplement: Supplement 1 [file media-1.pdf]

Supplementary Information

Discovery of 2-amide-3-methylester thiophenes that target SARS-CoV-2 Mac1 and repress coronavirus replication, validating Mac1 as an anti-viral target

Sarah Wazir[a,#], Tomi A. O. Parviainen[b,#], Jessica J. Pfannenstiel[c], Men Thi Hoai Duong[a], Daniel Cluff[c], Sven T. Sowa[a], Albert Galera-Prat[a], Dana Ferraris[d], Mirko M. Maksimainen[a], Anthony R. Fehr[c,*], Juha P. Heiskanen[b,*] & Lari Lehtiö[a,*]

[a]Faculty of Biochemistry and Molecular Medicine & Biocenter Oulu, University of Oulu, Finland

[b]Research Unit of Sustainable Chemistry, University of Oulu, P.O. Box 4300, FI-90014 Oulu, Finland

[c]Department of Molecular Biosciences, University of Kansas, Lawrence, Kansas, United States of America.

[d]McDaniel College Department of Chemistry, 2 College Hill, Westminster, MD, USA.

[#]These authors contributed equally

*Address correspondence to arfehr@ku.edu, Juha.Heiskanen@oulu.fi or Lari.Lehtio@oulu.fi

CONTENT

Figure S1: IC_{50} measurements of hit compounds.
Figure S2: Thermal shift assay.
Figure S3: Cell viability assays.
Figure S4: Comparison of Mac1 and MacroD2 crystal structures.
Table S1. Assay validation statistics.
Table S2. Hit validation and counter screening.
Table S3. Crystallography data and refinement statistics.
Figure S4-35. NMR spectra
Figure S36. HPLC chromatogram of **27** at 220nm detection wavelength.
Figure S37. HPLC chromatogram of **27** at 260nm detection wavelength.

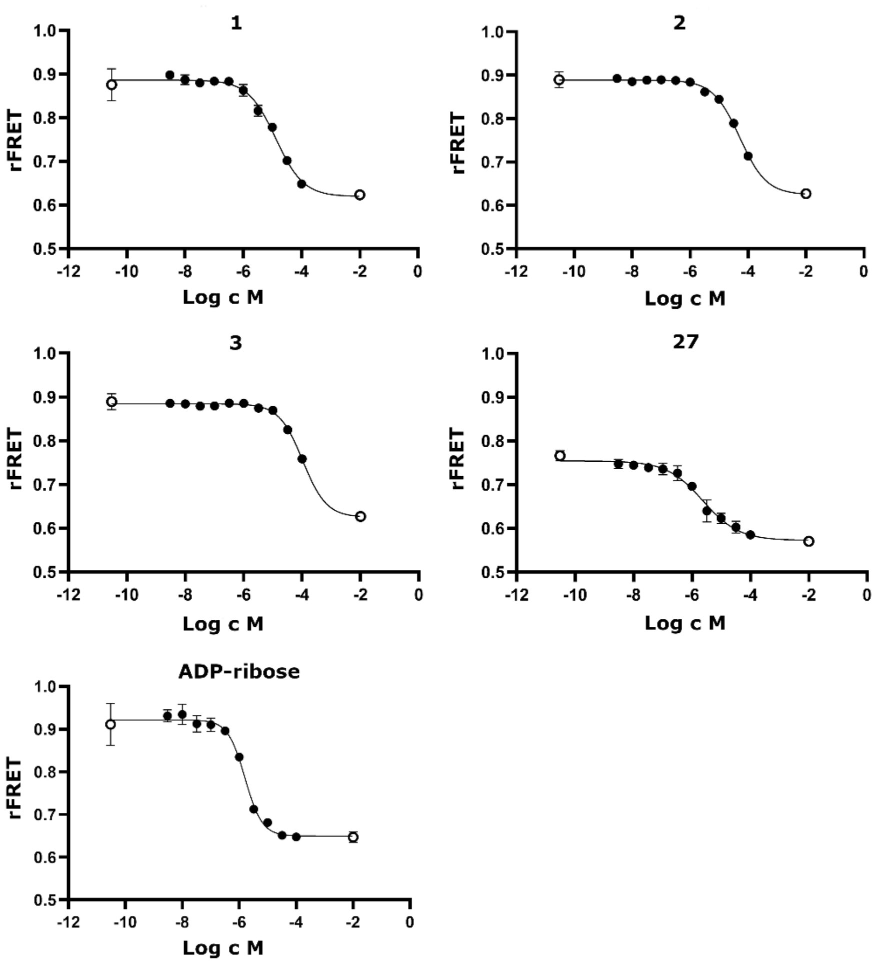

Figure S1. Representative IC_{50} measurements of SARS-CoV-2 Mac1 for ADPr and initial hit compounds. Measurements were carried out in half-logarithmic dilution starting from 100 to 0.003 µM for each curve. Negative control (containing no compound) was set one logarithmic unit below the lowest concentration. positive control (containing 200 µM ADPr) was set one logarithmic unit above the highest concentration of the compound. Data is reported as mean ± SD of four replicates.

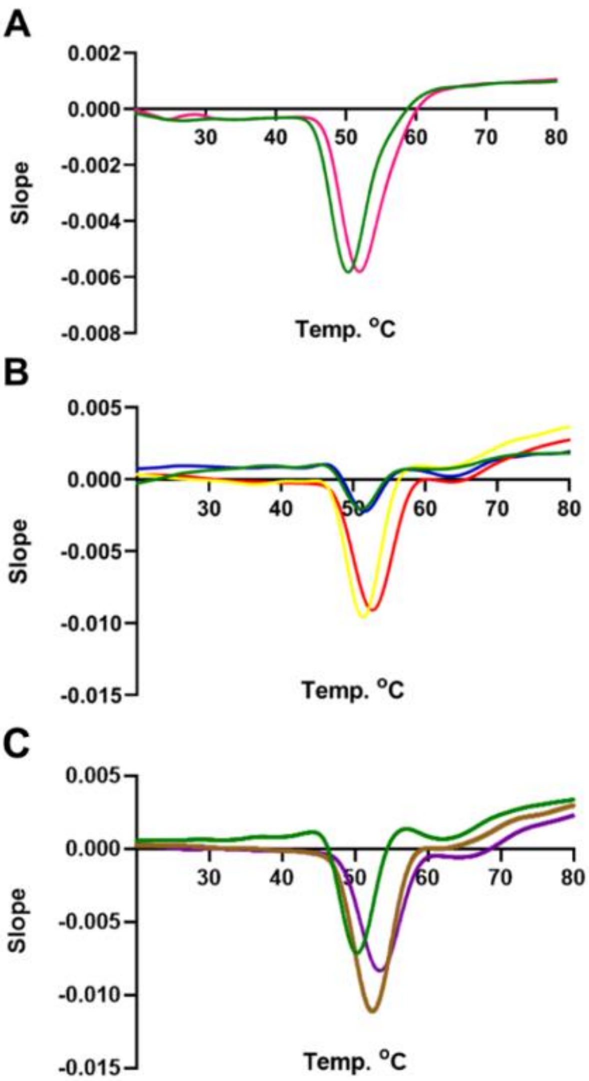

Figure S2. Thermal shift assay of SARS-CoV-2 Mac1 for ADPr and initial hit compounds and the most potent compound **27**. (A) buffer control (green). ADPr (pink) (B) DMSO control (green). **1** (Red). **2** (Yellow). **3** (blue) (C) DMSO control (green). 30 µM **27** (brown), 60 µM **27** (purple). Each curve was measured three times, and a representative second derivative curve is shown.

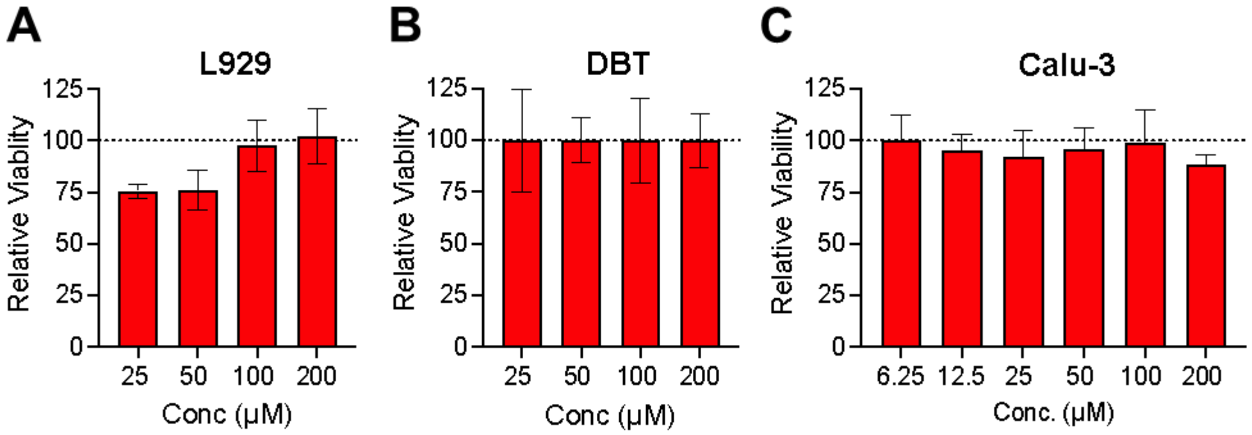

Figure S3. Compound **27** does not affect the viability of mouse or human cell lines. (A-B) L929 (A), DBT (B) and Calu-3 (C) cells were treated with compound **27** at the indicated concentrations for 24 hours and then viability was measured by an MTT assay and normalized to the viability level of DMSO treated cells. The results in A and B are representative of 3 independent experiments, while the results in C are the combined results of 3 independent experiments. n=3.

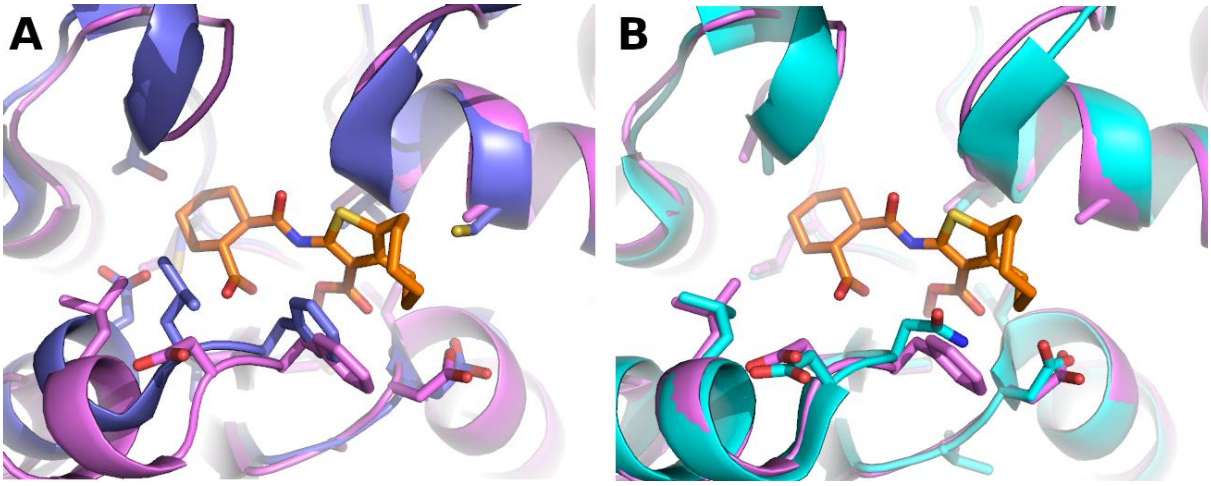

Figure S4. Comparison of macrodomain crystal structures. (A) Superimposed SARS-CoV-2 Mac1 in complex with **27** (PDB: 8TV7, orange) and MacroD2 (PDB: 6Y4Y, blue). (B) SARS-CoV-2 Mac1 in complex with **27** and SARS-CoV Mac1 (PDB: 2ACF, cyan). Binding site residues and **27** is shown in sticks (orange).

Table S1. Assay validation statistics.

Statistical parameters	FRET assay
S/B	1.33 ± 0.03
S/N	22.04 ± 2.48
Z'	0.81 ± 0.02
Well-to-well CV (max/min. %)	0.95 ± 0.22 / 0.82 ± 0.19
Plate-to-plate CV (%)*	0.33
Day-to-day CV (%)*	2.55

*Calculated from Z' values.

Table S2. Hit validation and counter screening results with TNKS ARC4 assay.

ID	Figure	TNKS2 ARC4	NanoDSF ΔTm °C	Inhibition % primary screening		IC_{50} (pIC_{50} ± SEM), n=3
				410 nm	430 nm	
**1**	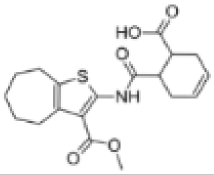	NI*	1.0	60.5	73.3	14 µM (-4.85 ± 0.02)
**2**	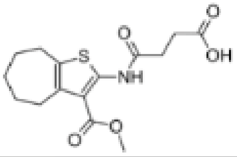	NI	0.5	23.3	40.6	48 µM (-4.32 ± 0.02)
**3**	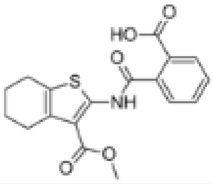	NI	1.0	29.2	31.8	110 µM (-3.96 ± 0.02)
**4**	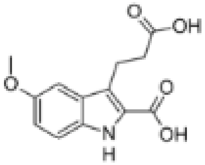	NI	0.3	37.6	39.5	89 µM (-4.05 ± 0.01)
**5**	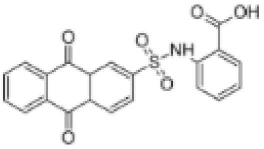	NI	0.1	32.3	30.5	45 µM (-4.34 ± 0.03)
**6**	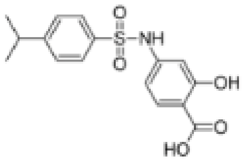	NI	1.0	36.0	48.9	57 µM (-4.24 ± 0.03)
**7**	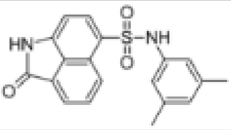	NI	0.1	64.5	59.7	NI
**8**	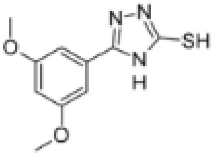	NI	0.3	75.5	77.5	57 µM (-4.26 ± 0.07)

*NI. no inhibition

Table S3. Data collection and refinement statistics for co-crystal structures.

**Inhibitor (PDB id.)**	**1 (8TV6)**	**27 (8TV7)**
**Data collection**		
Beamline	ID30A-1 ESRF	ID30A-1 ESRF
Wavelength (Å)	0.9655	0.9655
Space group	P1	$P2_12_12_1$
Unit cell dimensions		
a, *b*, *c* (Å)	30.20, 37.80, 64.10	38.0, 55.0, 71.9
α, β, γ (°)	95.60, 98.10, 90.40	90.0, 90.0, 90.0
Resolution range (Å)	37.58-1.74 (1.80-[illegible]	43.72-1.50 (1.53-1.50)
Total no. of reflections	28635 (27298)	132023 (10008)
No. of unique reflections	27365 (1499)	25009 (1245)
Completeness (%)	95.4 (94.6)	99.9 (99.9)
$I/\sigma(I)$	7.3 (2.1)	9.8 (1.7)
CC1/2 (%)	99.0 (76.0)	99.7 (64.1)
R_{meas}	10.5 (46.7)	11.5 (92.5)
**Model building and refinement**		
R-factor	17.4	17.0
R-free	22.4	19.0
No. of atoms		
Protein	2580	1363
Ligands*	46	39
Water	220	146
RMSD		
Bonds (Å)	0.0075	0.0124
Angles (°)	0.1441	0.1590
Average *B* factors (Å^2)	23.03	17.39
Protein	15.27	12.94
Ligands*	31.11	15.44
Water	22.73	23.80
Ramachandran plot		
Favoured (%)	100	94
Allowed (%)	0.0	6.0
Outliers (%)	0.0	0.0

[1]Values within parentheses refers to the highest resolution shell. *Inhibitor, PEG and glycerol molecules.

Figure S4. ^{1}H NMR spectrum of compound **9a** in $CDCl_3$ (-0.5–15 ppm).

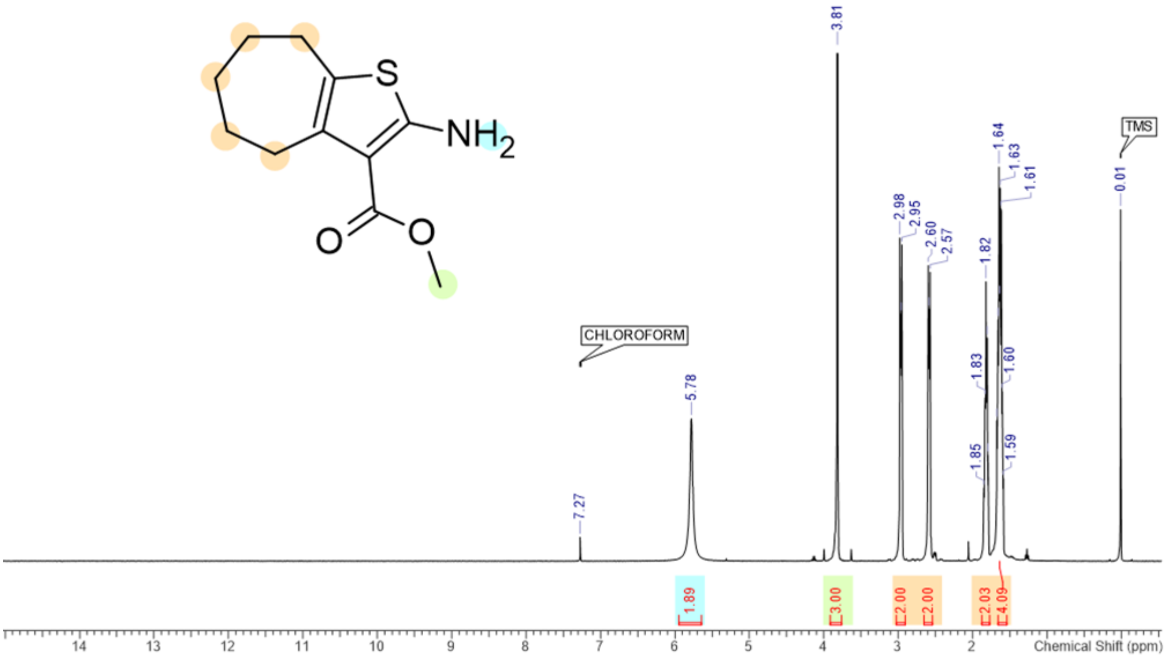

Figure S5. ^{1}H NMR spectrum of compound **9b** in $CDCl_3$ (-0.5–15 ppm).

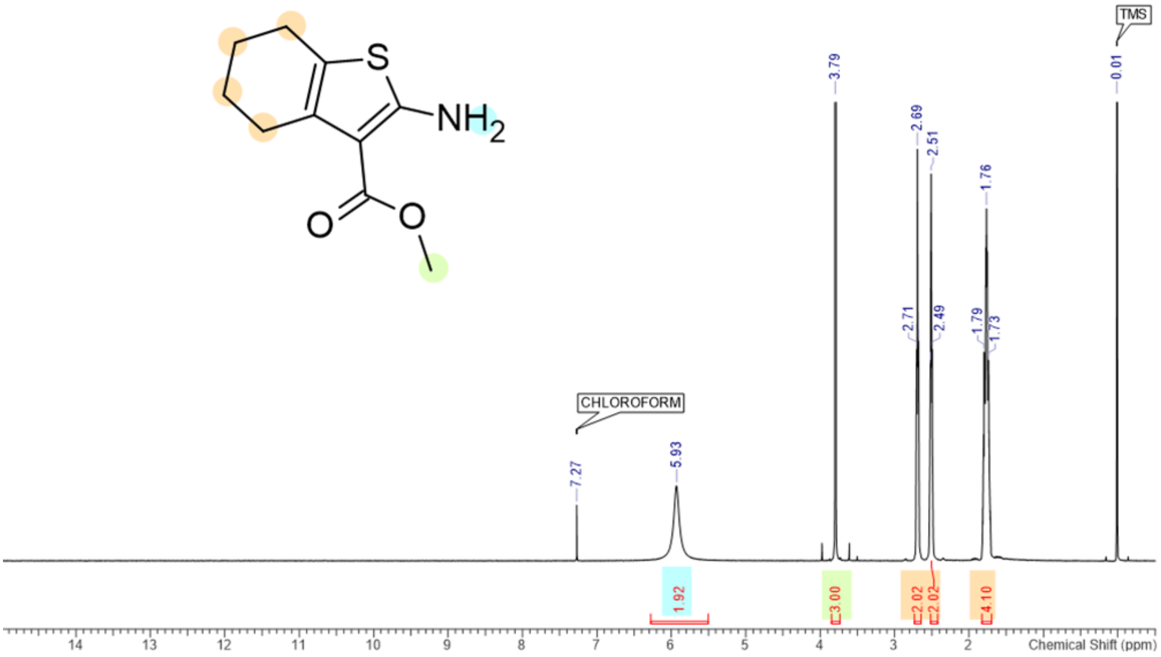

Figure S6. ^{1}H NMR spectrum of compound **9c** in $CDCl_3$ (-0.5–15 ppm).

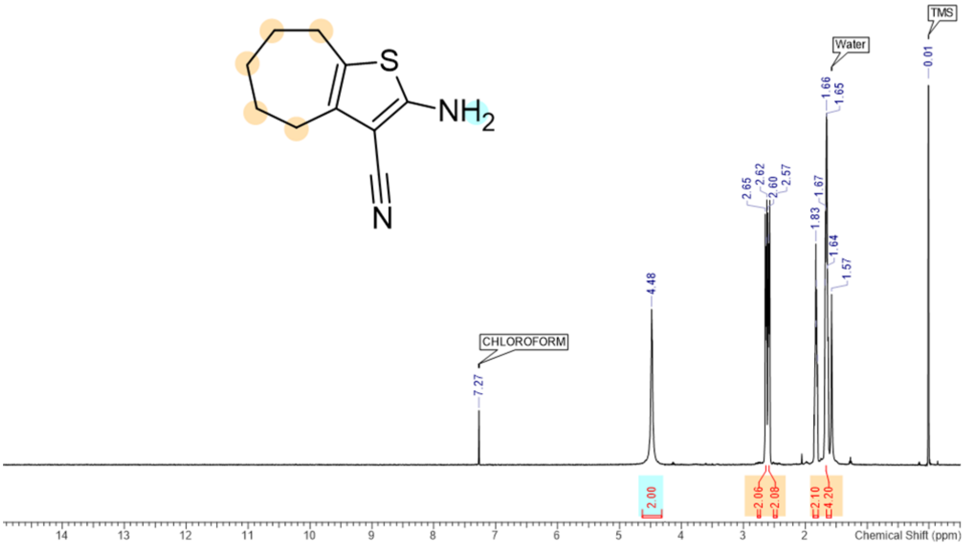

Figure S7. ^{1}H NMR spectrum of compound **9d** in $CDCl_3$ (-0.5–15 ppm).

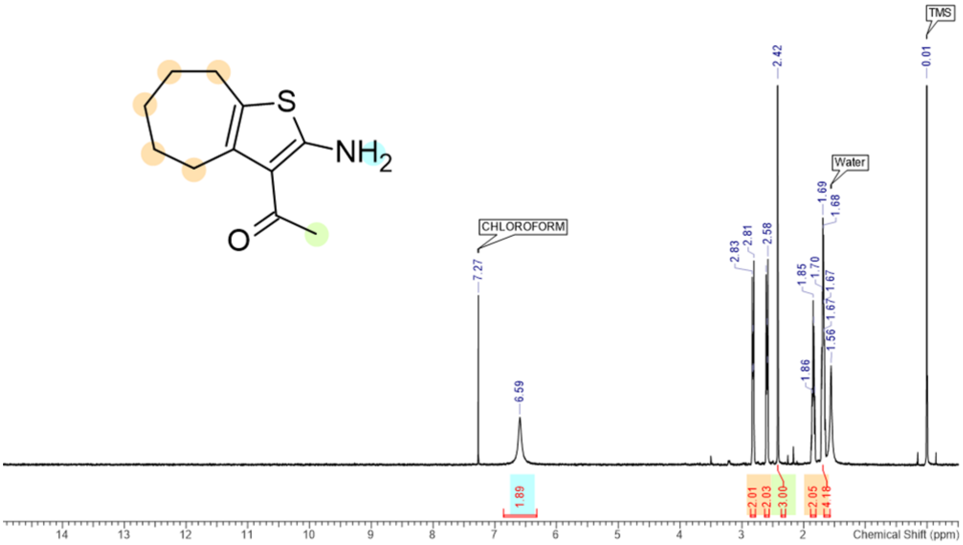

Figure S8. ^{1}H NMR spectrum of compound **9e** in d_6-DMSO (-0.5–15 ppm).

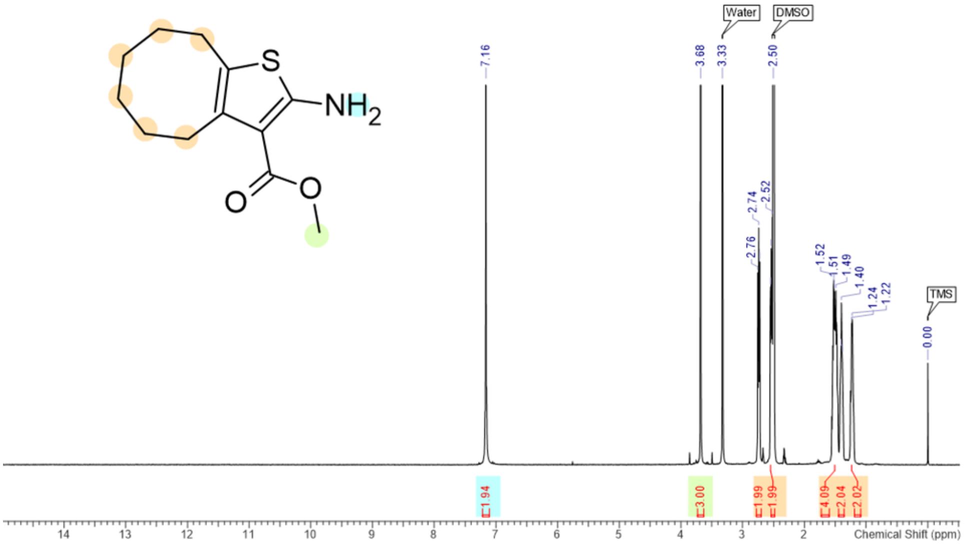

Figure S9. ^{1}H NMR spectrum of compound **1r** in $CDCl_3$ (-0.5–15 ppm).

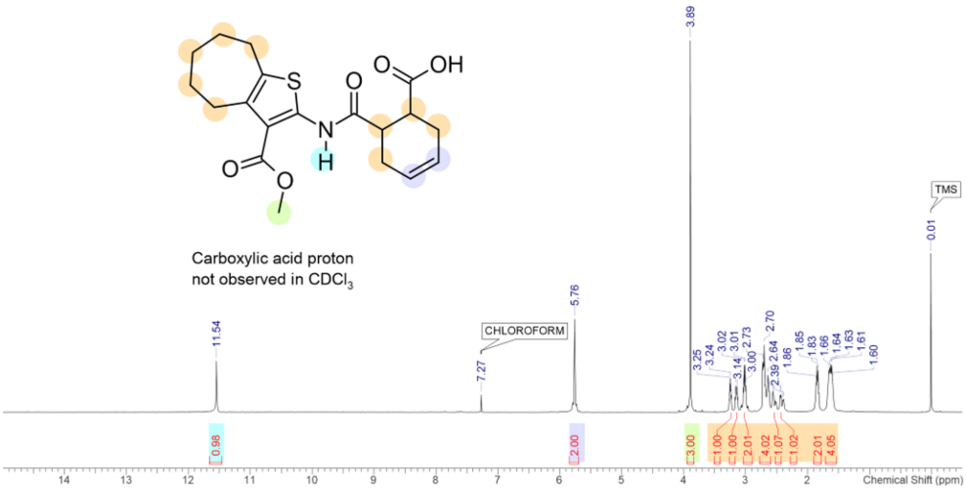

Figure S10. ^{1}H NMR spectrum of compound **1r** in d_6-DMSO (-0.5–15 ppm).

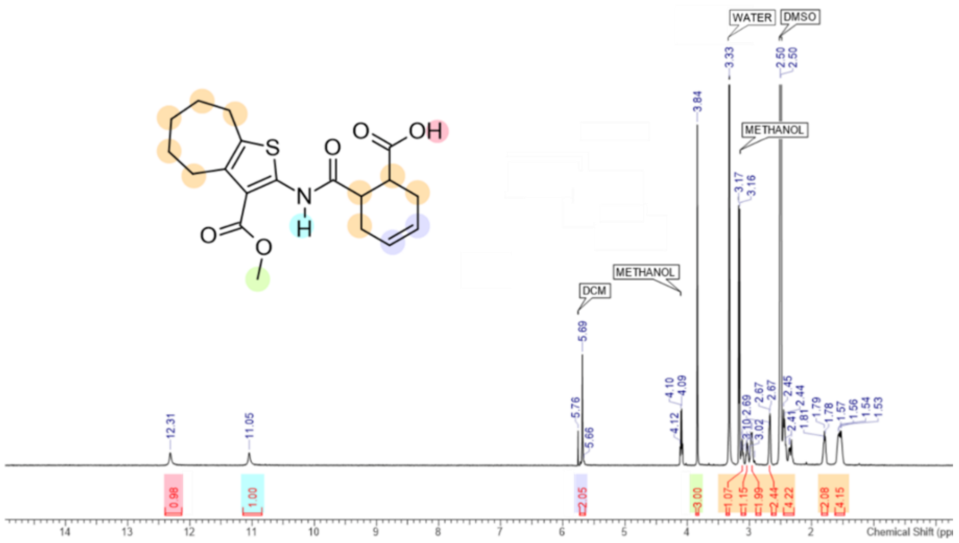

Figure S11. ^{13}C NMR spectrum of compound **1r** in $CDCl_3$ (0–220 ppm)

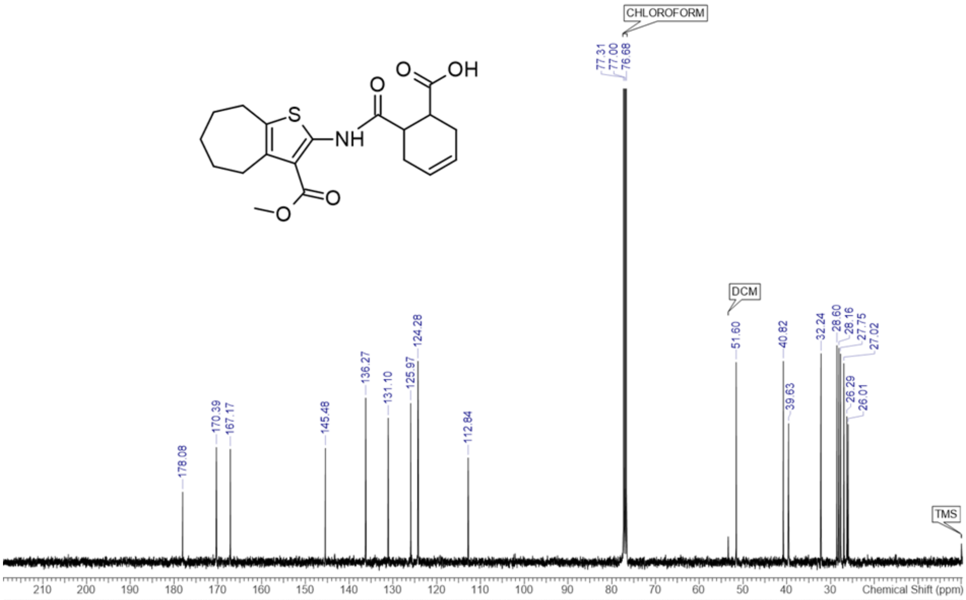

No.	(ppm)	(Hz)	Height	No.	(ppm)	(Hz)	Height	No.	(ppm)	(Hz)	Height
1	26.01	2617.2	0.1200	8	39.63	3987.5	0.1206	15	136.27	13710.6	0.1429
2	26.29	2645.0	0.1268	9	40.82	4107.1	0.1748	16	145.48	14637.1	0.0992
3	27.02	2718.4	0.1729	10	51.60	5192.1	0.1739	17	167.17	16819.6	0.0984
4	27.75	2791.7	0.1813	11	112.84	11352.8	0.0909	18	170.39	17143.1	0.1000
5	28.16	2833.6	0.1864	12	124.28	12503.8	0.1750	19	178.08	17917.0	0.0611
6	28.60	2877.6	0.1886	13	125.97	12674.0	0.1382				
7	32.24	3243.6	0.1815	14	131.10	13190.5	0.1255				

Figure S12. ^{1}H NMR spectrum of compound **3r** in d_6-DMSO (-0.5–15 ppm).

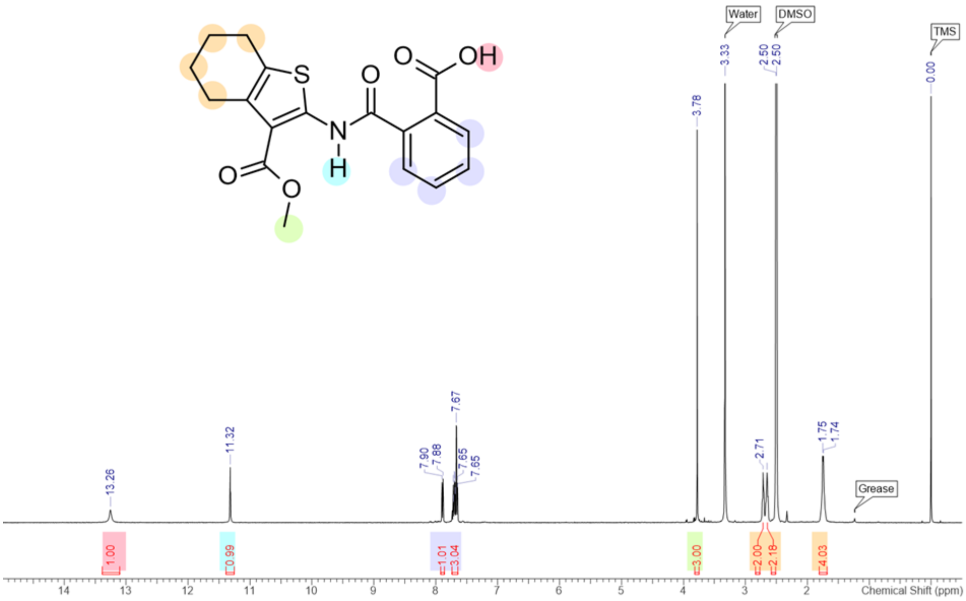

Figure S13. ^{13}C NMR spectrum of compound **3r** in $CDCl_3$ (0–220 ppm).

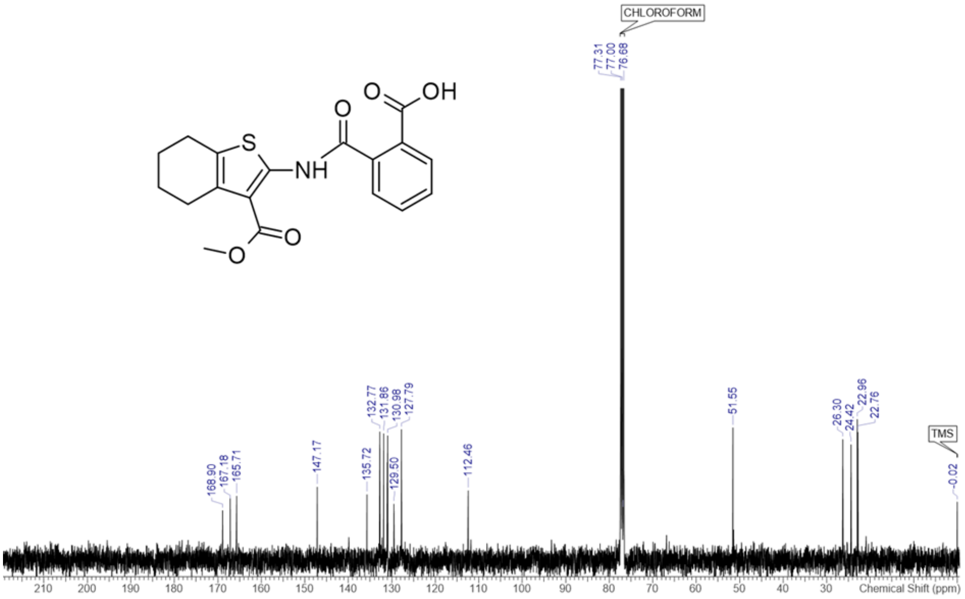

No.	(ppm)	(Hz)	Height	No.	(ppm)	(Hz)	Height	No.	(ppm)	(Hz)	Height
1	22.76	2290.0	0.0375	7	127.75	12853.7	0.0236	13	132.77	13358.5	0.0377
2	22.96	2309.8	0.0413	8	127.79	12857.4	0.0383	14	135.72	13655.6	0.0193
3	24.42	2456.5	0.0339	9	129.50	13029.1	0.0165	15	147.17	14807.3	0.0215
4	26.30	2645.8	0.0354	10	130.98	13178.0	0.0365	16	165.71	16672.1	0.0189
5	51.55	5186.9	0.0389	11	131.06	13186.8	0.0287	17	167.18	16820.3	0.0172
6	112.46	11314.7	0.0204	12	131.86	13266.8	0.0371	18	168.90	16993.4	0.0146

Figure S14. ^{1}H NMR spectrum of compound **10** in d_6-DMSO (-0.5–15 ppm).

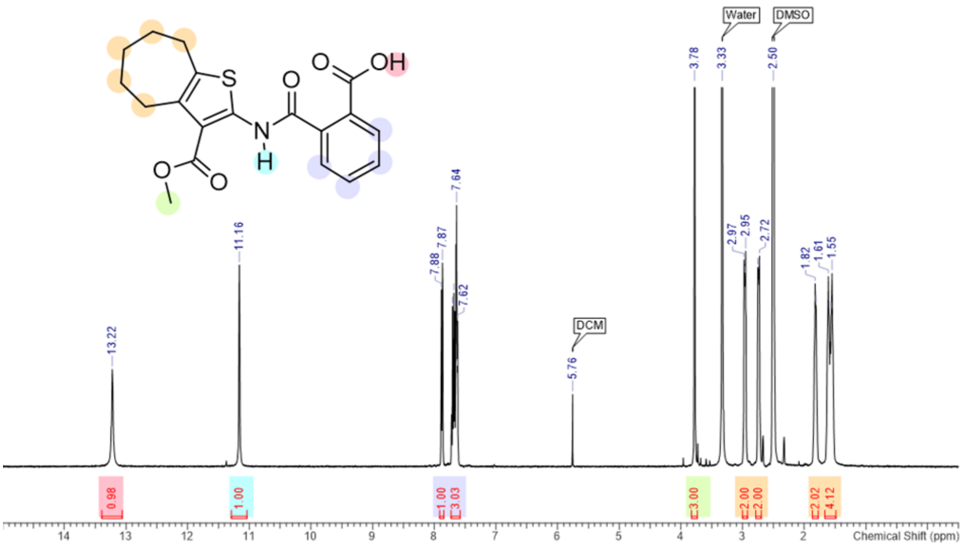

Figure S15. ^{13}C NMR spectrum of compound **10** in $CDCl_3$ (0–220 ppm).

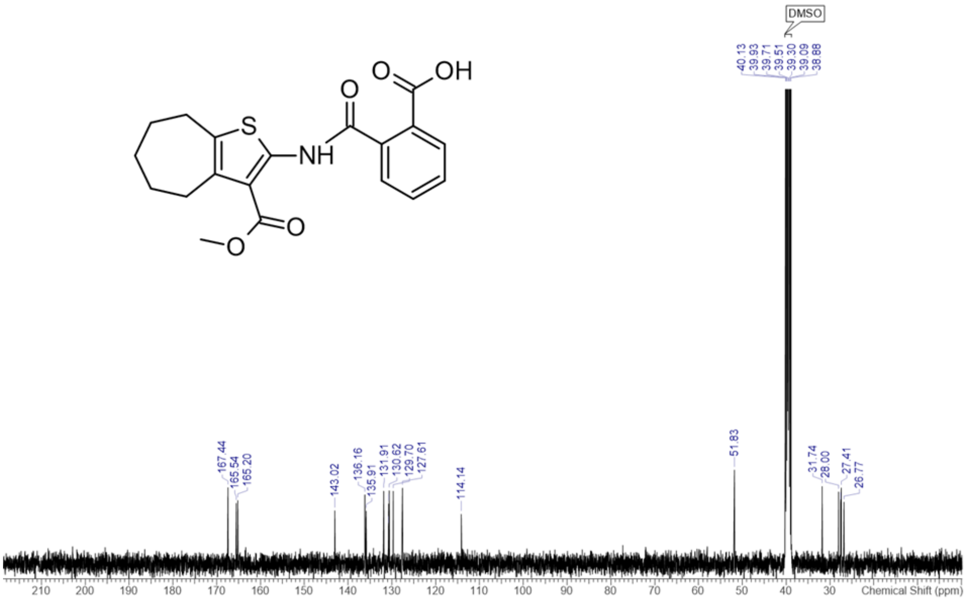

No.	(ppm)	(Hz)	Height	No.	(ppm)	(Hz)	Height	No.	(ppm)	(Hz)	Height
1	26.77	2693.6	0.0151	8	127.61	12839.2	0.0185	15	136.16	13699.8	0.0169
2	27.41	2757.4	0.0185	9	129.70	13049.8	0.0177	16	143.02	14389.3	0.0130
3	27.50	2767.0	0.0150	10	130.62	13142.2	0.0179	17	165.20	16620.9	0.0155
4	28.00	2816.9	0.0176	11	130.77	13156.9	0.0147	18	165.54	16655.4	0.0148
5	31.74	3193.2	0.0189	12	130.84	13164.2	0.0092	19	167.44	16846.9	0.0186
6	51.83	5214.3	0.0229	13	131.91	13272.1	0.0178				
7	114.14	11484.3	0.0122	14	135.91	13674.1	0.0129				

Figure S16. ^{1}H NMR spectrum of compound **11** in $CDCl_3$ (-0.5–15 ppm).

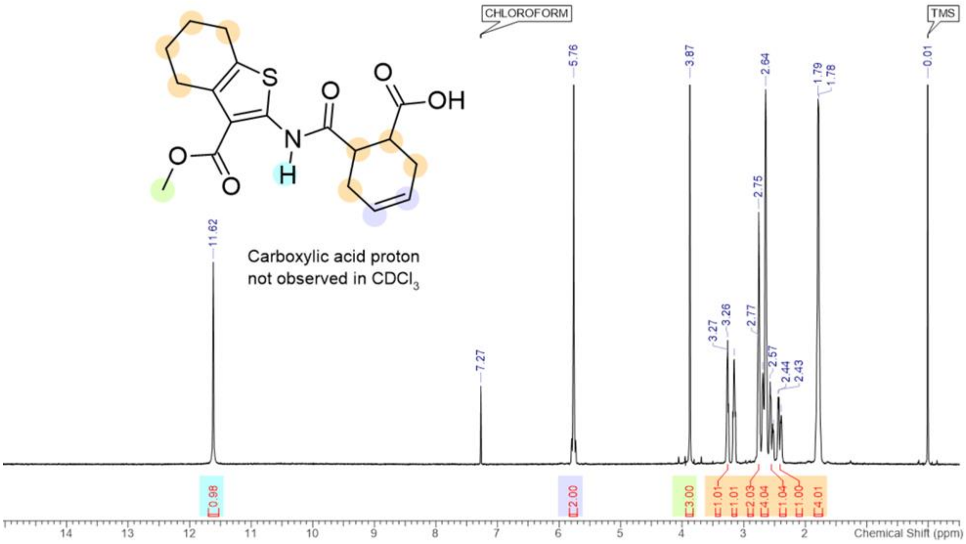

Figure S17. ^{13}C NMR spectrum of compound **11** in $CDCl_3$ (0–220 ppm).

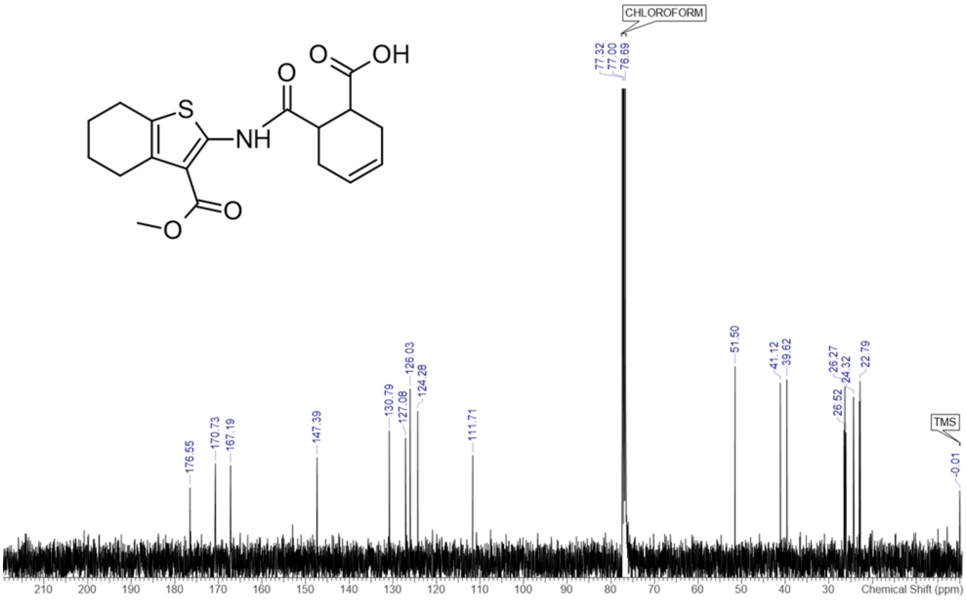

No.	(ppm)	(Hz)	Height	No.	(ppm)	(Hz)	Height	No.	(ppm)	(Hz)	Height
1	22.79	2292.9	0.0366	7	39.62	3986.8	0.0369	13	127.08	12786.3	0.0251
2	22.96	2309.8	0.0325	8	41.12	4137.2	0.0362	14	130.79	13158.9	0.0265
3	24.32	2447.0	0.0334	9	51.50	5181.8	0.0396	15	147.39	14829.3	0.0211
4	26.11	2626.7	0.0263	10	111.71	11239.1	0.0216	16	167.19	16821.0	0.0195
5	26.27	2643.6	0.0355	11	124.28	12504.6	0.0305	17	170.73	17177.6	0.0199
6	26.52	2667.8	0.0267	12	126.03	12679.9	0.0351	18	176.55	17763.0	0.0150

Figure S18. ^{1}H NMR spectrum of compound **15r** in d_6-DMSO (-0.5–15 ppm).

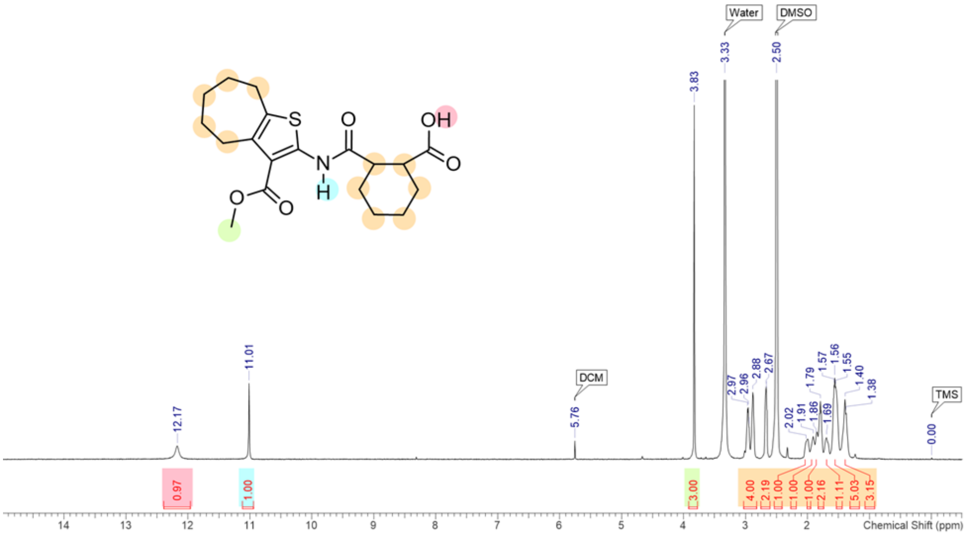

Figure S19. ^{13}C NMR spectrum of compound **15r** in d_6-DMSO (0–220 ppm).

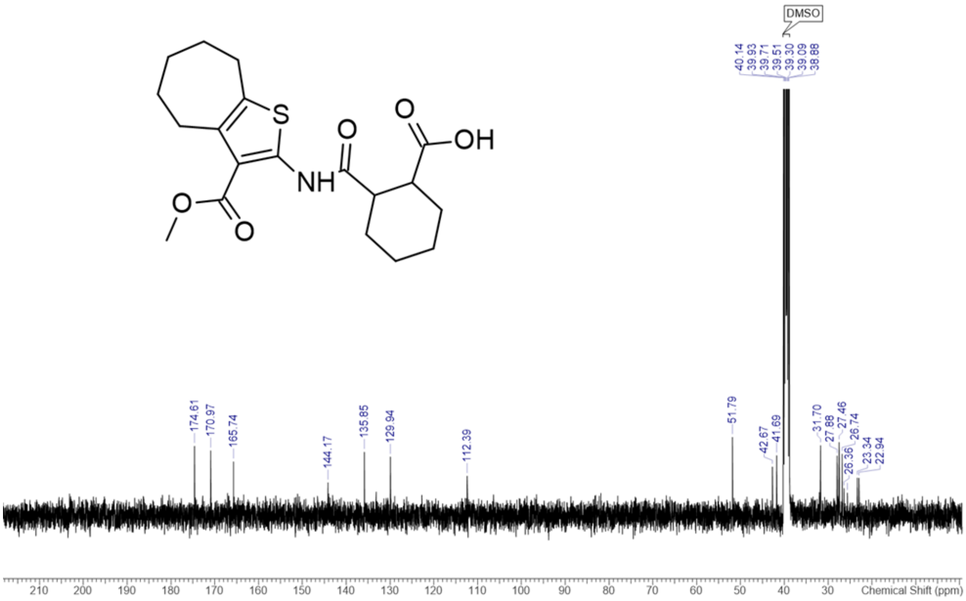

No.	(ppm)	(Hz)	Height	No.	(ppm)	(Hz)	Height	No.	(ppm)	(Hz)	Height
1	22.94	2307.8	0.0076	8	27.88	2805.1	0.0121	15	135.85	13668.2	0.0128
2	23.34	2348.1	0.0076	9	31.70	3189.5	0.0141	16	144.17	14505.2	0.0066
3	25.56	2571.8	0.0046	10	41.69	4194.6	0.0121	17	165.74	16675.2	0.0109
4	26.36	2651.8	0.0055	11	42.67	4292.9	0.0098	18	170.97	17201.9	0.0131
5	26.74	2690.0	0.0124	12	51.79	5210.6	0.0158	19	174.61	17568.0	0.0140
6	27.41	2758.2	0.0139	13	112.39	11308.2	0.0080				
7	27.46	2763.3	0.0148	14	129.94	13073.3	0.0118				

Figure S20. ^{1}H NMR spectrum of compound **21** in d_6-DMSO (-0.5–15 ppm).

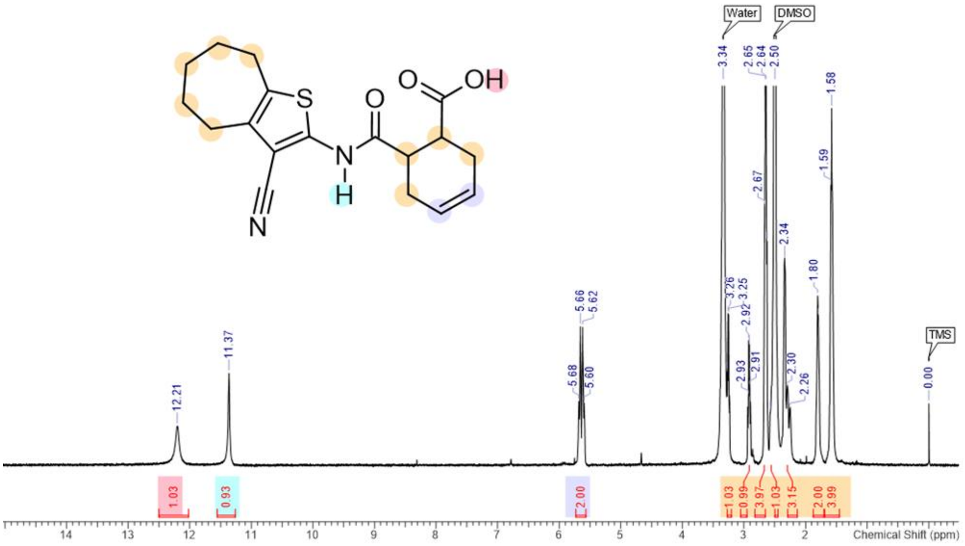

Figure S21. ^{13}C NMR spectrum of compound **21** in $CDCl_3$ (0–220 ppm).

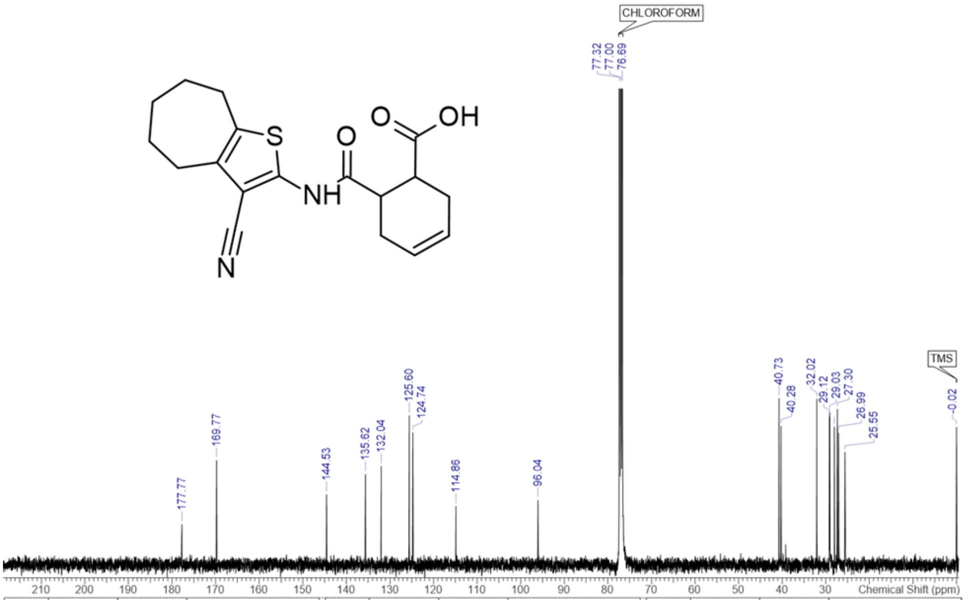

No.	(ppm)	(Hz)	Height	No.	(ppm)	(Hz)	Height	No.	(ppm)	(Hz)	Height
1	25.55	2570.9	0.0131	7	32.02	3221.6	0.0193	13	125.60	12636.6	0.0174
2	26.99	2715.5	0.0154	8	40.28	4052.8	0.0162	14	132.04	13285.1	0.0115
3	27.30	2747.0	0.0181	9	40.73	4098.3	0.0194	15	135.62	13645.3	0.0105
4	28.00	2817.4	0.0161	10	96.04	9662.6	0.0075	16	144.53	14541.7	0.0082
5	29.03	2920.9	0.0178	11	114.86	11556.0	0.0068	17	169.77	17081.5	0.0122
6	29.12	2929.7	0.0173	12	124.74	12550.0	0.0154	18	177.77	17886.2	0.0047

Figure S22. ^{1}H NMR spectrum of compound **22** in d_6-DMSO (-0.5–15 ppm).

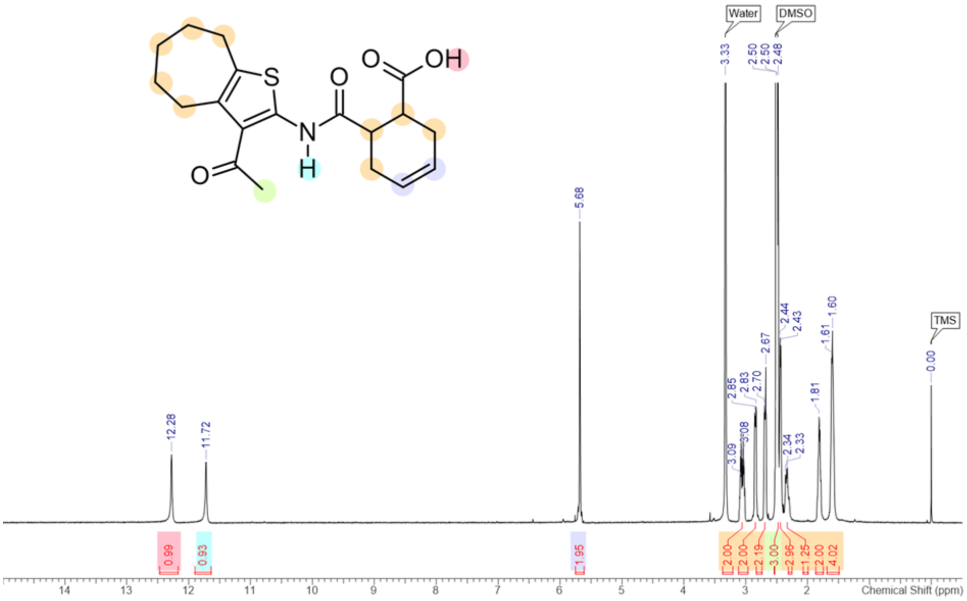

Figure S23. ^{13}C NMR spectrum of compound **22** in $CDCl_3$ (0–220 ppm).

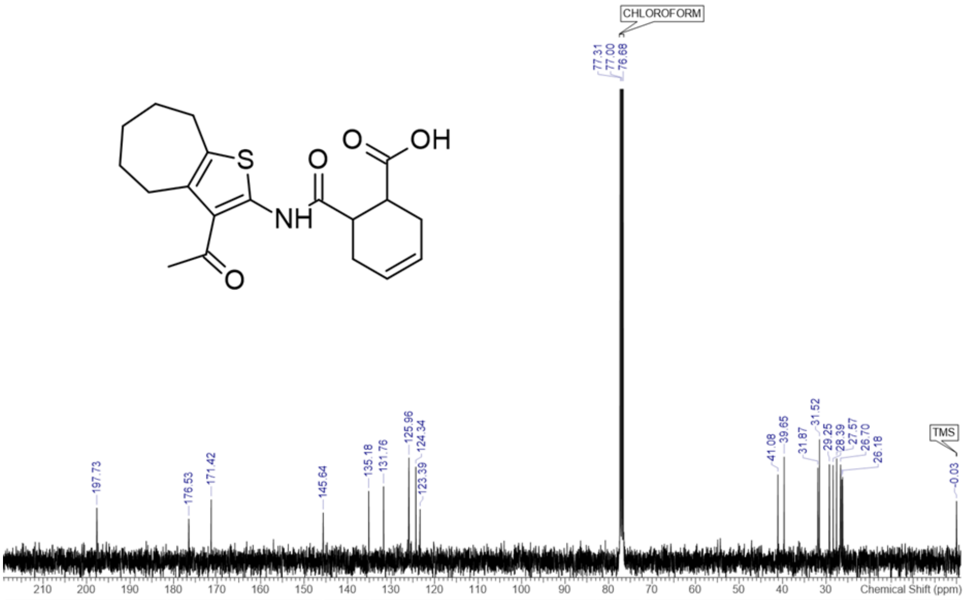

No.	(ppm)	(Hz)	Height	No.	(ppm)	(Hz)	Height	No.	(ppm)	(Hz)	Height
1	26.18	2634.0	0.0296	8	31.87	3206.2	0.0328	15	135.18	13600.5	0.0246
2	26.35	2651.6	0.0287	9	39.65	3989.0	0.0366	16	145.64	14653.3	0.0170
3	26.70	2686.1	0.0339	10	41.08	4133.5	0.0304	17	171.42	17247.2	0.0216
4	27.57	2774.1	0.0360	11	123.39	12415.1	0.0182	18	176.53	17761.5	0.0149
5	28.39	2856.3	0.0337	12	124.34	12510.4	0.0331	19	197.73	19894.1	0.0187
6	29.25	2942.9	0.0340	13	125.96	12673.3	0.0363				
7	31.52	3171.0	0.0427	14	131.76	13256.5	0.0262				

Figure S24. ^{1}H NMR spectrum of compound **23** in d_6-DMSO (-0.5–15 ppm).

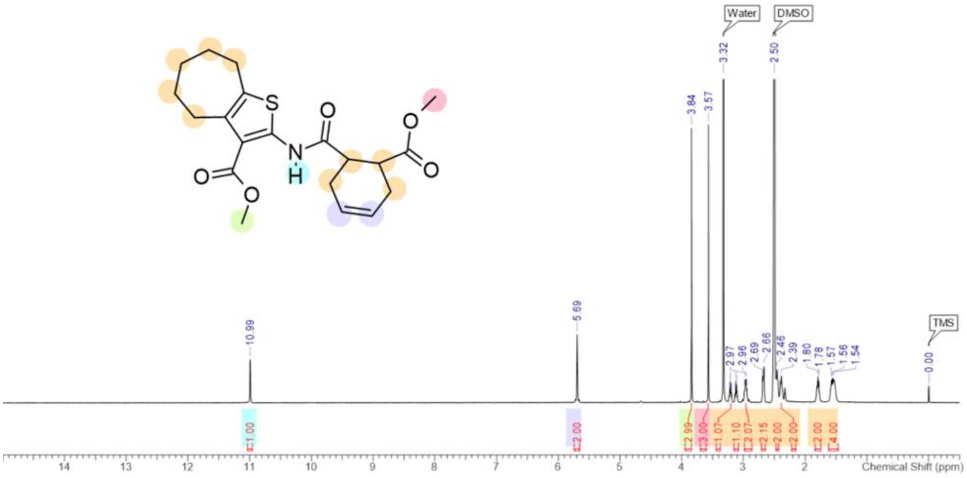

Figure S25. ^{13}C NMR spectrum of compound **23** in $CDCl_3$ (0–220 ppm).

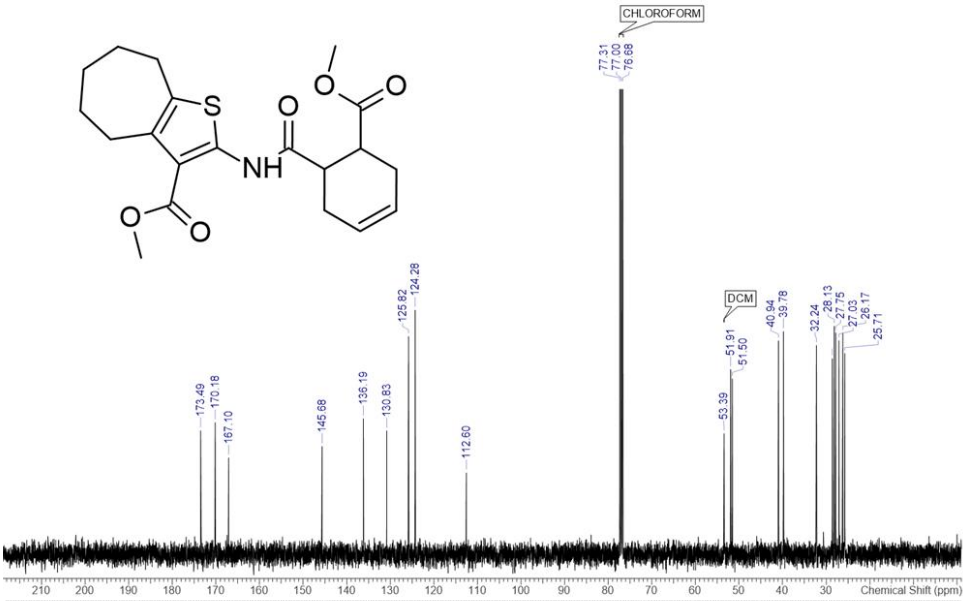

No.	(ppm)	(Hz)	Height	No.	(ppm)	(Hz)	Height	No.	(ppm)	(Hz)	Height
1	25.71	2587.1	0.2566	8	39.78	4002.2	0.2846	15	130.83	13163.3	0.1580
2	26.17	2633.3	0.2826	9	40.94	4119.6	0.2728	16	136.19	13702.5	0.1734
3	27.03	2719.1	0.2730	10	51.50	5181.8	0.2241	17	145.68	14657.7	0.1380
4	27.75	2792.5	0.2829	11	51.91	5222.9	0.2361	18	167.10	16812.2	0.1233
5	28.13	2830.6	0.2914	12	112.60	11328.6	0.1042	19	170.18	17122.5	0.1684
6	28.58	2875.4	0.2500	13	124.28	12503.8	0.3119	20	173.49	17455.6	0.1581
7	32.24	3243.6	0.2669	14	125.82	12659.3	0.2782				

Figure S26. ^{1}H NMR spectrum of compound **24** in d_6-DMSO (-0.5–15 ppm).

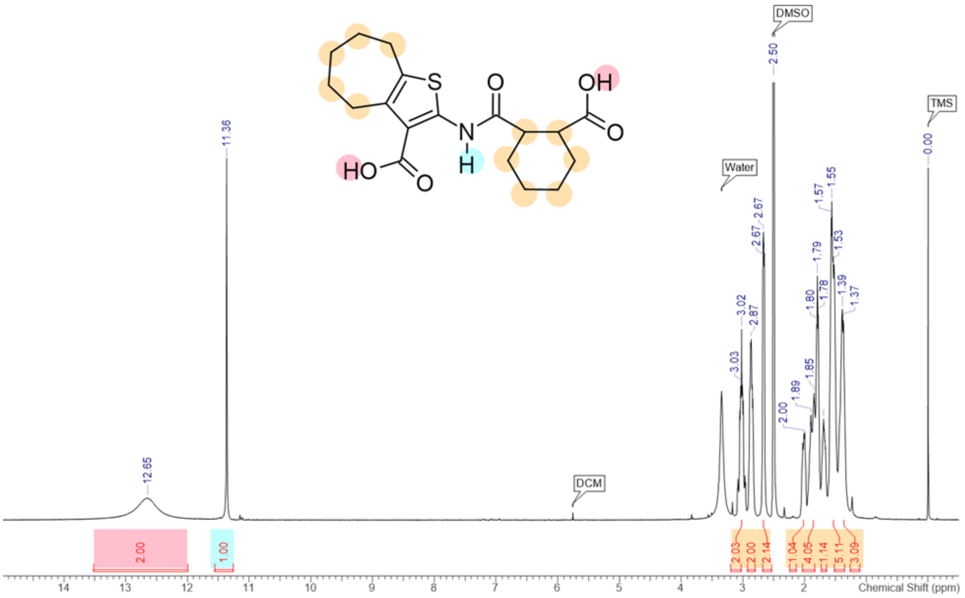

Figure S27. ^{13}C NMR spectrum of compound **24** in d_6-DMSO (0–220 ppm).

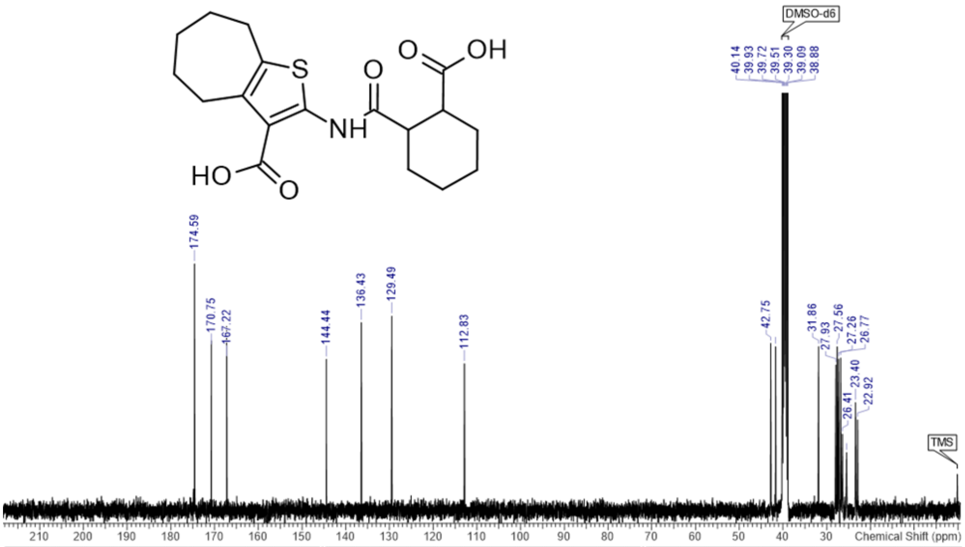

No.	(ppm)	(Hz)	Height	No.	(ppm)	(Hz)	Height	No.	(ppm)	(Hz)	Height
1	22.92	2305.5	0.0250	7	27.56	2772.8	0.0452	13	129.49	13028.5	0.0536
2	23.40	2354.0	0.0298	8	27.93	2810.3	0.0401	14	136.43	13726.2	0.0518
3	25.43	2558.6	0.0160	9	31.86	3205.7	0.0452	15	144.44	14532.4	0.0417
4	26.41	2657.7	0.0211	10	41.66	4191.6	0.0452	16	167.22	16824.1	0.0418
5	26.77	2693.6	0.0421	11	42.75	4301.7	0.0461	17	170.75	17179.9	0.0450
6	27.26	2742.8	0.0418	12	112.83	11352.2	0.0405	18	174.59	17565.8	0.0680

Figure S28. ^{1}H NMR spectrum of compound **25** in $CDCl_3$ (-0.5–15 ppm).

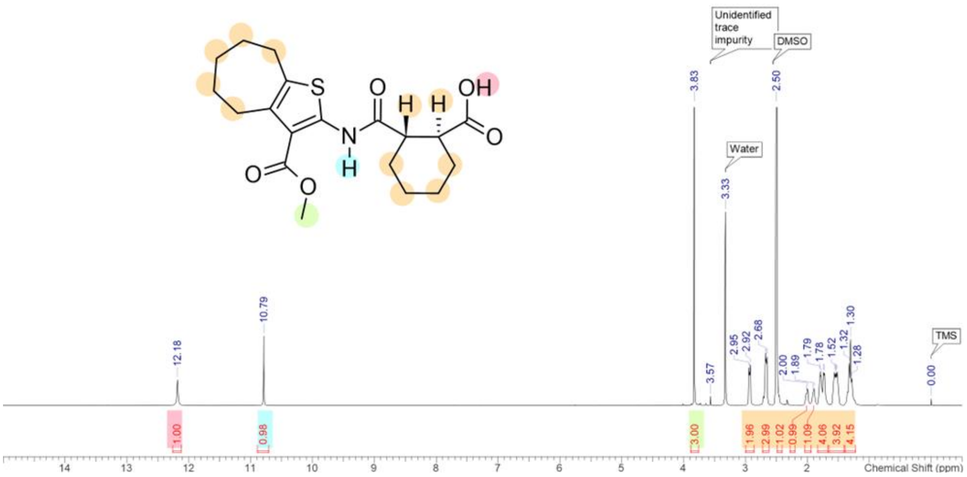

Figure S29. ^{13}C NMR spectrum of compound **25** in $CDCl_3$ (0–220 ppm).

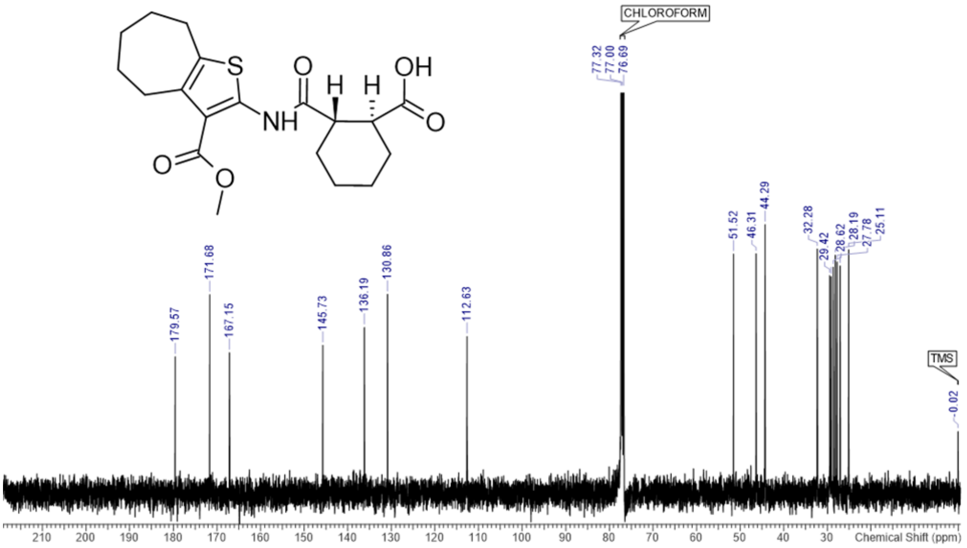

No.	(ppm)	(Hz)	Height	No.	(ppm)	(Hz)	Height	No.	(ppm)	(Hz)	Height
1	25.07	2522.5	0.0516	8	29.42	2960.5	0.0501	15	136.19	13702.5	0.0382
2	25.11	2526.2	0.0561	9	32.28	3247.3	0.0562	16	145.73	14662.8	0.0341
3	27.05	2722.1	0.0524	10	44.29	4456.3	0.0619	17	167.15	16817.4	0.0324
4	27.78	2795.4	0.0533	11	46.31	4659.5	0.0552	18	171.68	17273.7	0.0458
5	28.19	2836.5	0.0548	12	51.52	5184.0	0.0551	19	179.57	18067.4	0.0315
6	28.62	2879.0	0.0521	13	112.63	11332.3	0.0362				
7	29.12	2929.7	0.0499	14	130.86	13166.3	0.0458				

Figure S30. ^{1}H NMR spectrum of compound **26** in d_6-DMSO (-0.5–15 ppm).

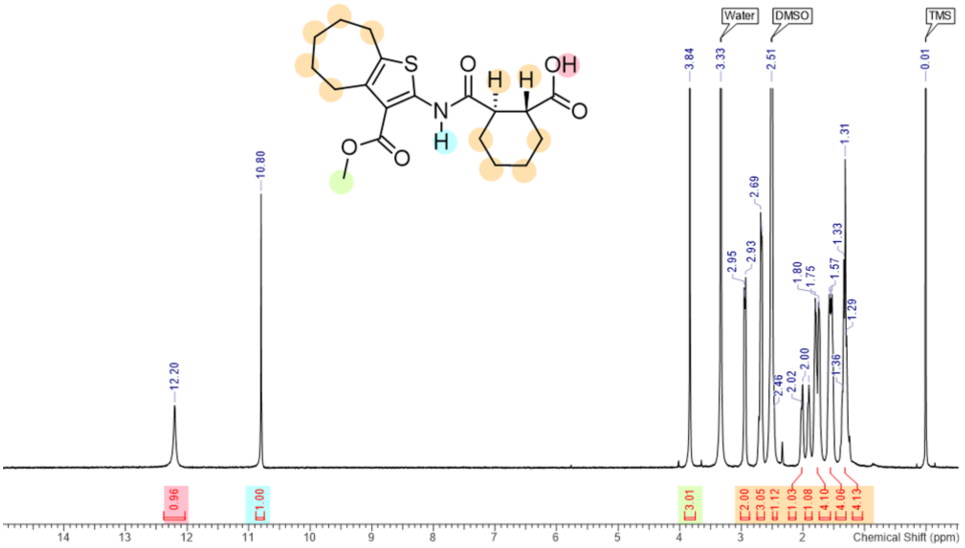

Figure S31. ^{13}C NMR spectrum of compound **26** in $CDCl_3$ (0–220 ppm).

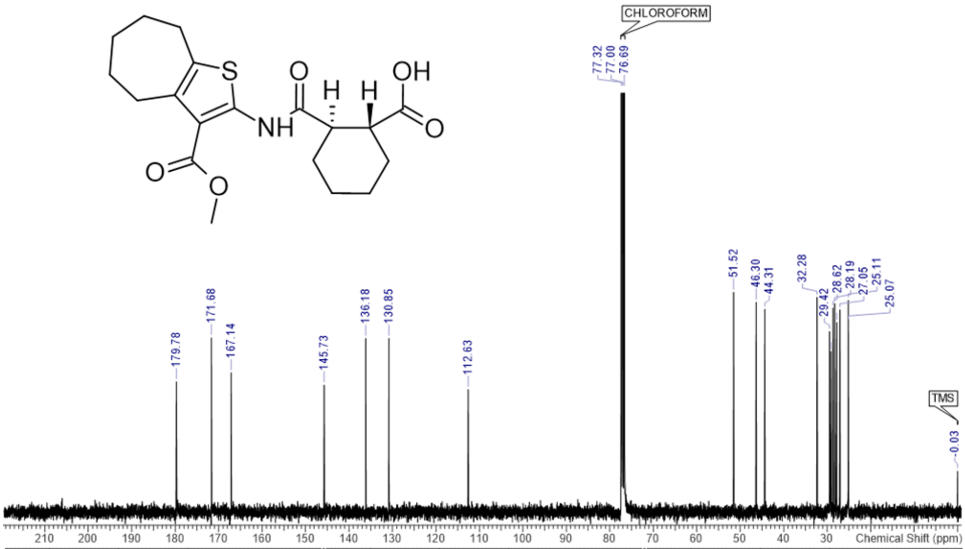

No.	(ppm)	(Hz)	Height
1	25.07	2522.5	0.0751
2	25.11	2526.2	0.0837
3	27.05	2722.1	0.0799
4	27.78	2795.4	0.0749
5	28.19	2836.5	0.0824
6	28.62	2879.0	0.0806
7	29.13	2930.4	0.0635
8	29.42	2960.5	0.0713
9	32.28	3247.3	0.0849
10	44.31	4458.5	0.0801
11	46.30	4658.0	0.0829
12	51.52	5183.3	0.0869
13	112.63	11331.5	0.0485
14	130.85	13165.5	0.0686
15	136.18	13701.8	0.0686
16	145.73	14662.8	0.0502
17	167.14	16816.6	0.0551
18	171.68	17273.7	0.0688
19	179.78	18088.0	0.0515

Figure S32. ^{1}H NMR spectrum of compound **27** in d_6-DMSO (-0.5–15 ppm).

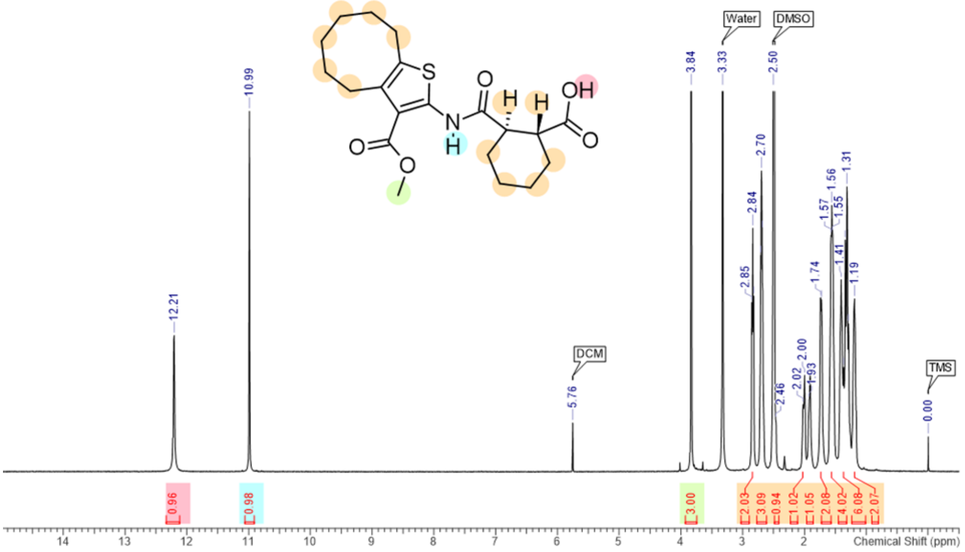

Figure S33. ^{13}C NMR spectrum of compound **27** in $CDCl_3$ (0–220 ppm)

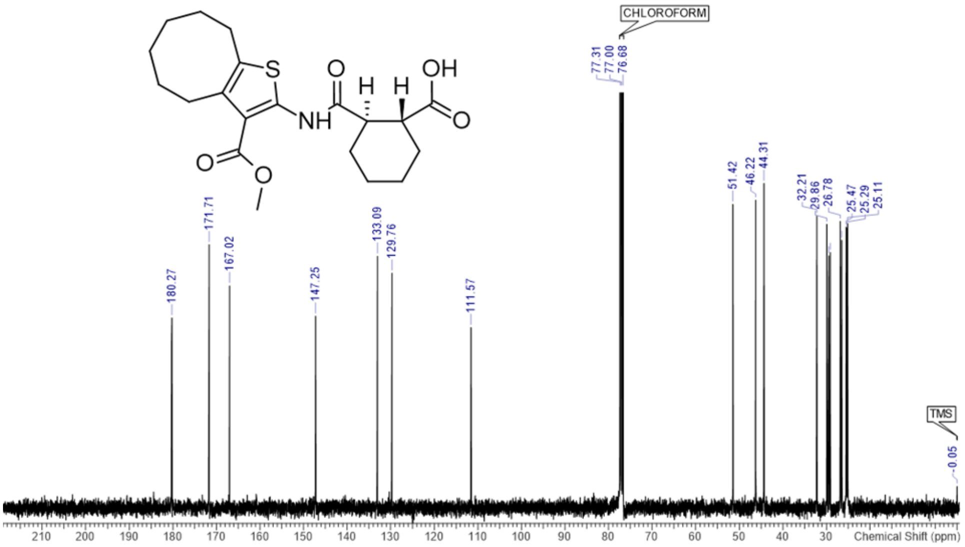

No.	(ppm)	(Hz)	Height	No.	(ppm)	(Hz)	Height	No.	(ppm)	(Hz)	Height
1	25.06	2521.8	0.1889	8	29.45	2962.7	0.1737	15	129.76	13055.5	0.1619
2	25.11	2526.2	0.1960	9	29.86	3004.5	0.1955	16	133.09	13390.7	0.1735
3	25.29	2544.5	0.1928	10	32.21	3240.7	0.2013	17	147.25	14815.4	0.1322
4	25.47	2562.1	0.1935	11	44.31	4457.7	0.2237	18	167.02	16804.2	0.1531
5	26.46	2661.9	0.1845	12	46.22	4649.9	0.2121	19	171.71	17276.6	0.1815
6	26.78	2694.9	0.1975	13	51.42	5173.7	0.2092	20	180.27	18137.8	0.1310
7	29.12	2929.7	0.1760	14	111.57	11225.2	0.1244				

Figure S34. ^{1}H NMR spectrum of compound **28** in $CDCl_3$ (-0.5–15 ppm).

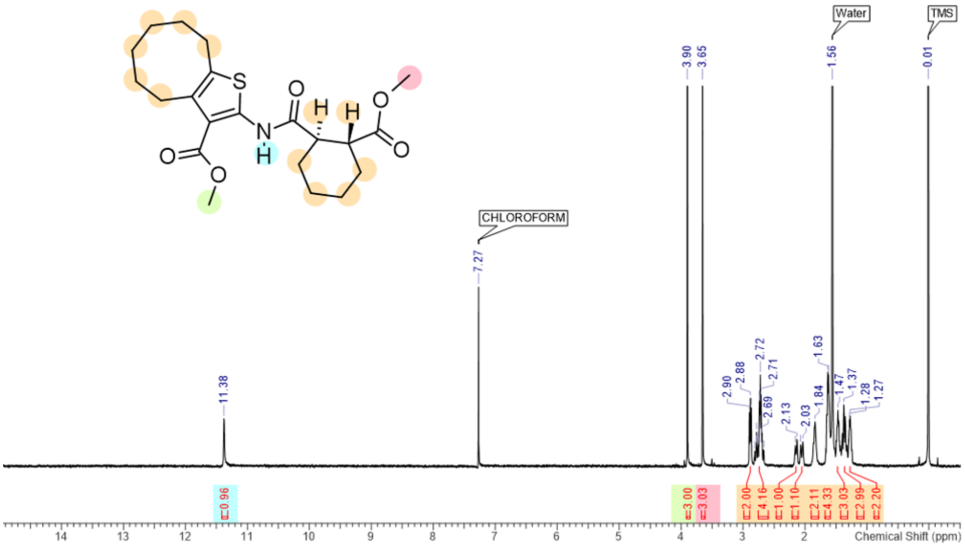

Figure S35. ^{13}C NMR spectrum of compound **28** in $CDCl_3$ (0–220 ppm).

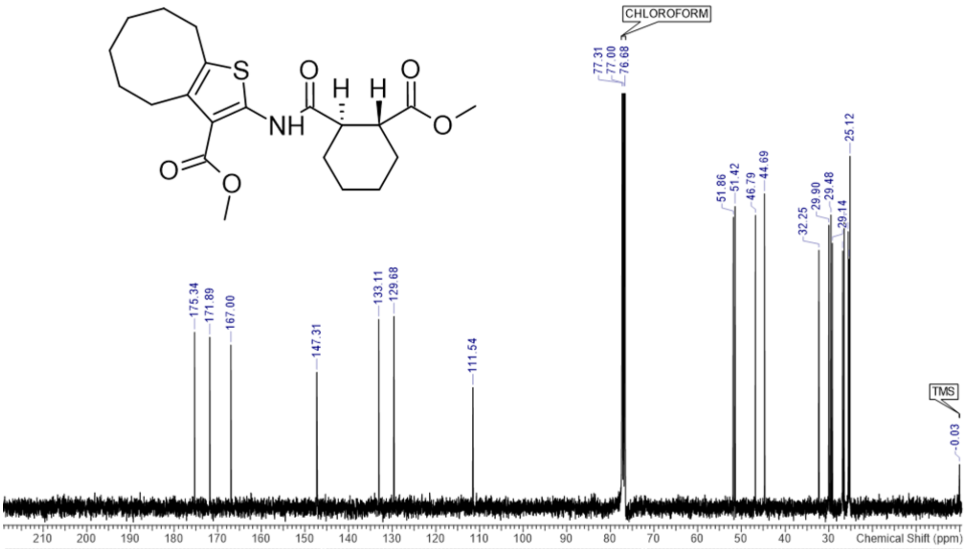

No.	(ppm)	(Hz)	Height	No.	(ppm)	(Hz)	Height	No.	(ppm)	(Hz)	Height
1	25.10	2525.5	0.1164	8	29.48	2965.6	0.1158	15	111.54	11222.2	0.0475
2	25.12	2527.7	0.1390	9	29.90	3008.2	0.1117	16	129.68	13047.4	0.0756
3	25.30	2546.0	0.0983	10	32.25	3245.1	0.1018	17	133.11	13392.9	0.0744
4	25.47	2562.1	0.1092	11	44.69	4495.9	0.1241	18	147.31	14821.2	0.0535
5	26.46	2661.9	0.1135	12	46.79	4707.9	0.1156	19	167.00	16802.7	0.0643
6	26.80	2696.4	0.1015	13	51.42	5173.7	0.1191	20	171.89	17294.2	0.0674
7	29.14	2931.9	0.1045	14	51.86	5217.7	0.1148	21	175.34	17641.9	0.0694

Figure S36. HPLC chromatogram of **27** at 220nm detection wavelength.

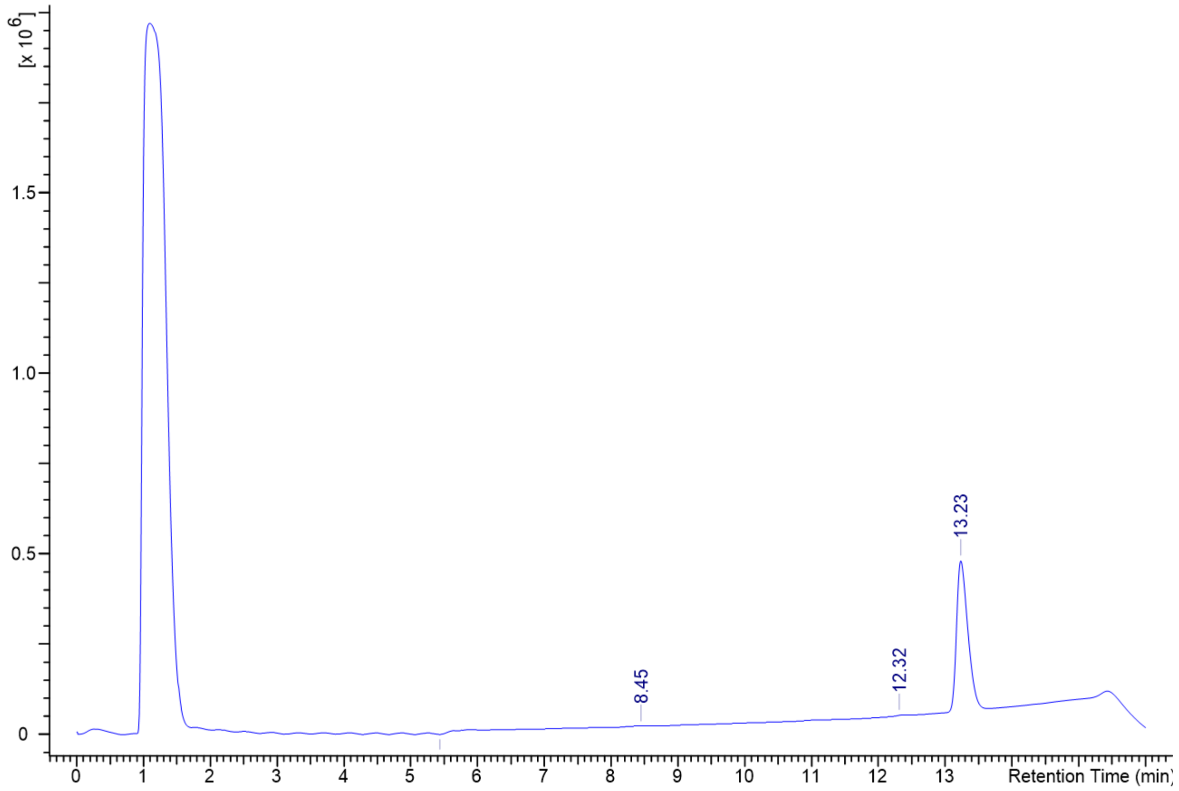

No.	tR	Peak Area (Y units*ms)	Area Percent	Width
1	8.45	36641216.000	0.739	0.650
2	12.32	16795072.000	0.339	0.179
3	13.23	4902276096.0(	98.922	0.308

Figure S37. HPLC chromatogram of **27** at 260nm detection wavelength.

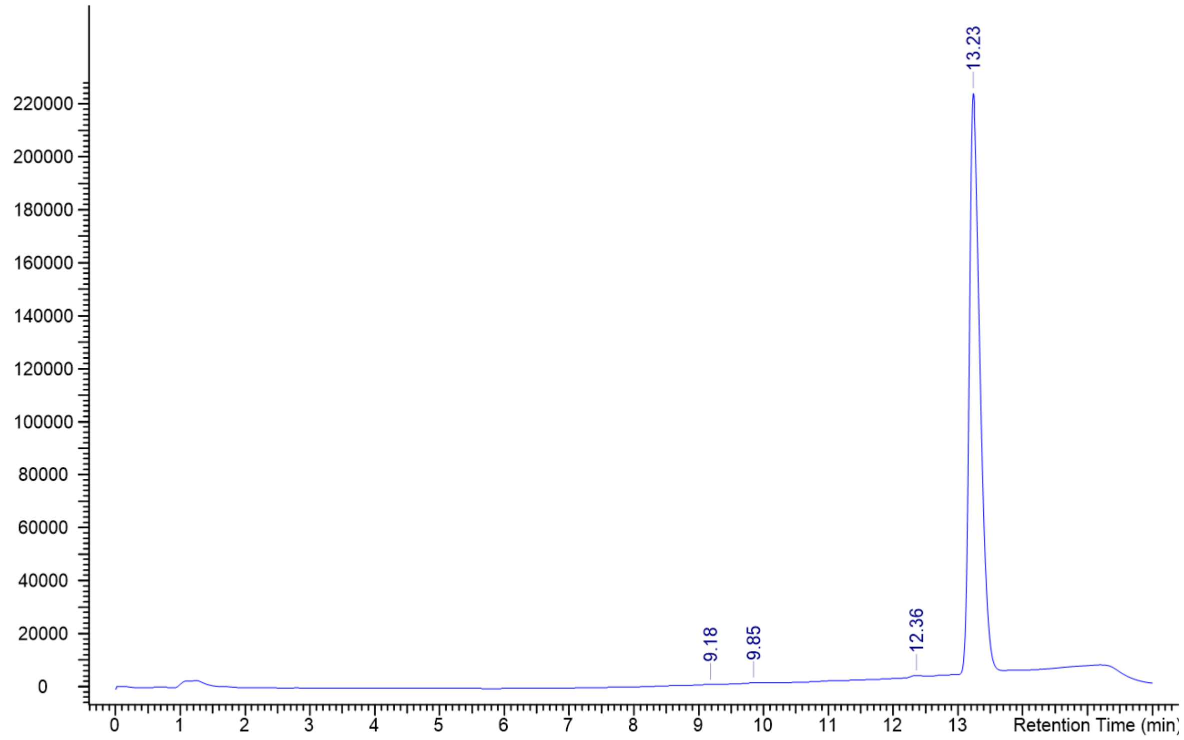

No.	tR	Peak Area (Y units*ms)	Area Percent	Width
1	9.18	4049237.000	0.156	0.456
2	9.85	7347586.500	0.283	0.790
3	12.36	6199013.500	0.238	0.287
4	13.23	2582017024.0(	99.323	0.309
